# Design and evaluation of primers targeting genes encoding NO-forming nitrite reductases: implications for ecological inference of denitrifying communities

**DOI:** 10.1038/srep39208

**Published:** 2016-12-14

**Authors:** Germán Bonilla-Rosso, Lea Wittorf, Christopher M. Jones, Sara Hallin

**Affiliations:** 1 Swedish University of Agricultural Sciences, Department of Forest Mycology and Plant Pathology, P.O. 7026, 75007 Uppsala, Sweden

## Abstract

The detection of NO-forming nitrite reductase genes (*nir*) has become the standard when studying denitrifying communities in the environment, despite well-known amplification biases in available primers. We review the performance of 35 published and 121 newly designed primers targeting the *nirS* and *nirK* genes, against sequences from complete genomes and 47 metagenomes from three major habitats where denitrification is important. There were no optimal universal primer pairs for either gene, although published primers targeting *nirS* displayed up to 75% coverage. The alternative is clade-specific primers, which show a trade-off between coverage and specificity. The test against metagenomic datasets showed a distinct performance of primers across habitats. The implications of clade-specific *nir* primers choice and their performance for ecological inference when used for quantitative estimates and in sequenced-based community ecology studies are discussed and our phylogenomic primer evaluation can be used as a reference along with their environmental specificity as a guide for primer selection. Based on our results, we also propose a general framework for primer evaluation that emphasizes the testing of coverage and phylogenetic range using full-length sequences from complete genomes, as well as accounting for environmental range using metagenomes. This framework serves as a guideline to simplify primer performance comparisons while explicitly addressing the limitations and biases of the primers evaluated.

Microorganisms carry out the cycling of inorganic nitrogen, which includes numerous reactions catalyzed by different enzymes present in a diverse range of bacterial, archaeal and fungal taxa. Nitrite reduction is a key step in the nitrogen cycle and is considered a branching point due to its role in several N-cycle pathways^1^. The reduction of nitrite can be a detoxification process in some organisms^2^, while in others it is part of energy conservation as in the processes denitrification (including nitrifier denitrification^3^) and anaerobic ammonia oxidation^4^. Moreover, nitrite reductases are involved in assimilatory and respiratory ammonification^1^. Of particular importance are microbial communities that perform nitrogen reduction pathways that result in the formation of gaseous products, since these processes account for net removal of available nitrogen from the ecosystem. While this leads to unwanted loss of applied N from soils, as well as the formation of the greenhouse gas nitrous oxide from subsequent steps in various N-cycle pathways, the removal of total nitrogen is essential in applications such as wastewater treatment.

Taxonomic molecular markers like the 16S rRNA gene do not provide sufficient information to capture the functional ecology of microbial communities at a detailed level, particularly when studying processes such as nitrite reduction as part of the denitrification pathway^5^. Instead, the key genes *nirS* and *nirK*, coding for the nitric oxide (NO)-forming nitrite (NO_2_^−^) reductases NirS and NirK respectively, are commonly used as molecular markers for denitrifying communities, and to a lesser extent for anammox since more specific molecular targets exist^6^. Although NirS and NirK both catalyze the reduction of NO_2_^−^ to NO, they are two non-homologous proteins that differ significantly in their evolution^5^, resulting in contrasting taxonomic^7^ and environmental distributions^8^,^9^,^10^. While metagenomics is often heralded as a means for functional microbial ecology, the low number of specific marker genes commonly detected in complex communities^11^ prevents comprehensive diversity estimates or community studies of particular functional guilds, thus making PCR-based approaches indispensable for the foreseeable future in studies of denitrifying and other functional communities. Yet, the usefulness of amplicon sequencing of *nir* genes and other functional genes relies heavily on both the existence of extensively tested primers targeting universally conserved regions, and on the availability of comprehensive, reliable and curated reference databases.

Primers targeting *nirS* and *nirK* were first designed from the few sequences available in the late 1990’s^12^,^13^, and reevaluated and updated more than ten years ago^14^. Since the *nir* genes arose early in evolution there is a high substitutional load in their sequences that makes primer design difficult. Consequently, primer bias and non-specificity have been pointed out recurrently^15^,^16^,^17^. In addition, analyses of *nir* genes obtained from genomes have shown that commonly used primer sets do not cover poorly studied clades within their phylogenies^5^, which impacts the interpretation of results from environmental studies into an ecological context. In an attempt to circumvent this problem, Wei and collaborators^18^ recently developed primer variants that target individual *nir* sequence clades, similar to what was done for the *nosZ* gene encoding the nitrous oxide reductase^19^. Although this greatly simplifies primer design, the usage of clade-specific primers brings additional considerations that need to be addressed due to the demand of such primer sets. A critical evaluation of the work by Wei *et al*.^18^ reveals that the primers designed in their study were based on sequence sets with reduced diversity that lacks well known clades (e.g. archaea in *nirS*; Thaumarchaea, Eukarya and Firmicutes in *nirK*^7^), resulting in inaccurate estimates of primer specificity and coverage. Even more disconcerting is the lack of a clear definition of evolutionarily conserved clades that is necessary for ecological inference based on the detection and quantification of specific *nir* clades. Prompted by this, we designed novel primer sets and performed updates on the specificity and inclusiveness of the existing sets. In addition, we review the usage of clade-specific primers for *nir* genes in denitrification and discuss problems with the interpretation of results using clade-specific primers, both for quantification of *nir* genes and analysis of diversity and composition of denitrifiers. Our approach includes an *in silico* analysis of 35 available *nirS* and *nirK* primers as well as 121 new primers designed in this study and was facilitated by the recent publication of robust and comprehensive phylogenies for both genes^7^. We used both fully sequenced genomes and 47 metagenomes to evaluate phylogenetic coverage and specificity as well as performance against environmental sequences lacking PCR-amplification bias.

## Results

### Phylogenetic coverage and specificity

**Figure 1:**
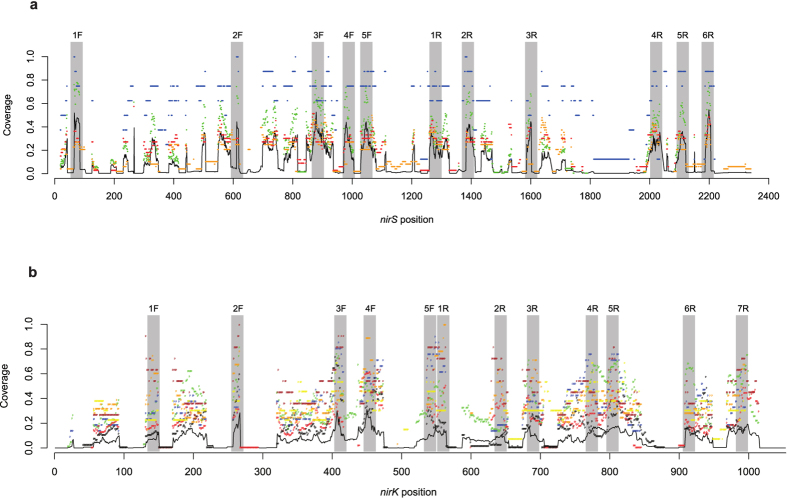
Sequence coverage plots for aligned full-length sequences obtained from complete microbial genomes. (**a**) *nirS* and (**b**) *nirK*. Plots show the proportion of sequences hit at every position in the alignment with a window length of 18 bp and a maximum degeneracy of 64. The black line indicates coverage across all sequences while points represent the clade-specific coverage based on the phylogenies presented in Fig. 3, with point color corresponding to clade labels within each phylogeny. The shaded gray boxes indicate conserved regions (>0.6) used for primer design, with corresponding labels for each region.

**Figure 2:**
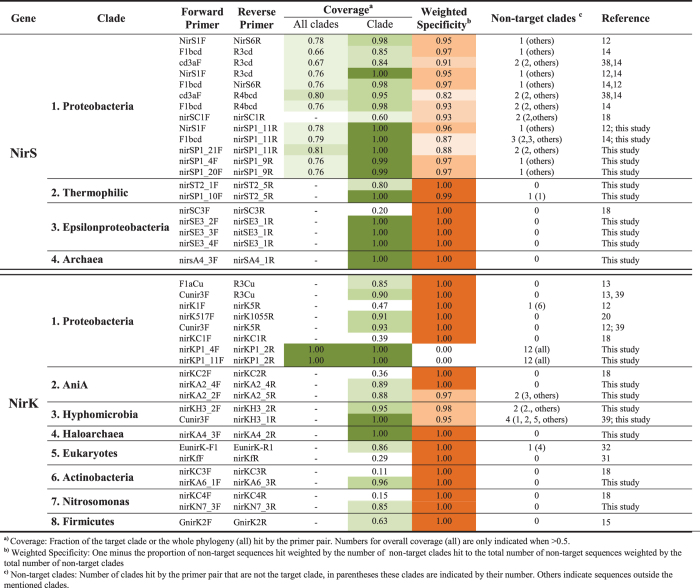
Primer performance in full-length sequences from complete genomes from Graf *et al*.^7^. The most commonly used primer pairs and the best of those designed in this study according to results in Tables S2 and S3 are shown. Performance was calculated from the number of sequences hit with a maximum of three mismatches using the “probe match” function in ARB^38^,^39^.

With the newly designed primers proposed here and the previously published primers, we evaluated 77 *nirS* and 79 *nirK* primers (Table S1). We did not identify any novel target regions in the alignments, and the sequence coverage profiles revealed that all possible conserved regions have already been used before for primer design (Fig. 1). None of the previously published primers designed to target each of the genes across all clades achieved complete coverage across genome-derived sequences, although the *nirS* primers performed relatively well (average coverage across all clades are 0.74 ± 0.05 for *nirS* vs. 0.17 ± 0.06 for *nirK;* Fig. 2). Only one primer pair designed in this study to target sequences in all *nirS* clades (nirSP1_21 F and nirSP1_11 R) displayed a higher coverage than previously published primers, whereas those for *nirK* only did so with prohibitively high degeneracy (Fig. 2). This underscores the problem with designing primers to cover a broad range of *nir* gene sequence diversity.

**Figure 3:**
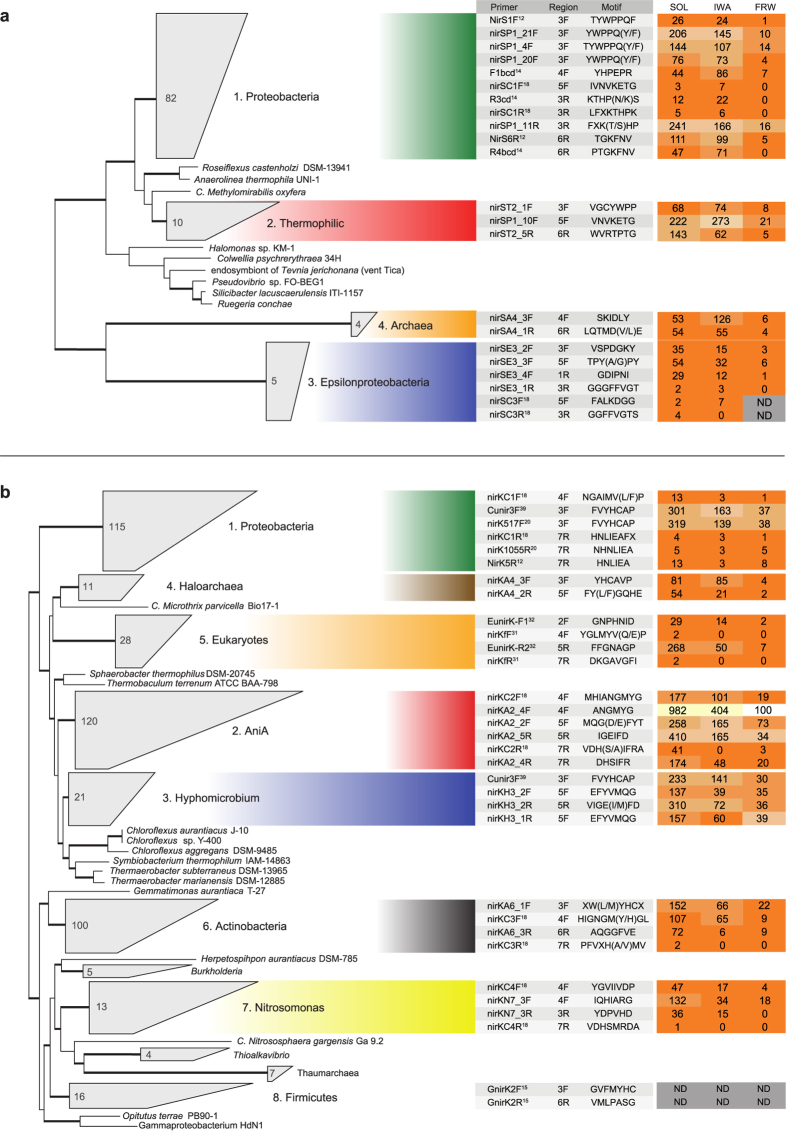
Performance of individual clade-specific primers against metagenomes. Previously published and best performing (i.e. highest within-clade coverage and specificity) primers from this study for (**a**) *nirS* and (**b**) *nirK*. The reference phylogenies are based on Graf *et al*.^7^ and the number of sequences within collapsed clades and branches with >65% bootstrap support are indicated on the trees. To the right, the names of primers found in each conserved region of the corresponding clades are shown together with the targeted amino acid motif. The references for previously published primers are indicated and the rest were designed in the present study. The heatmap shows the weighted specificity of each primer against reads from 47 metagenomes from soil (SOL), industrial wastewater (IWA) and freshwater (FRW), and the numbers correspond to the number of reads from non-target clades hit in each environment. (ND, no reads detected in that environment).

For the clade-specific primers, we did not find any published primer set that detects clades of archaeal *nirS* and *nirK*, or the thermophilic clade within the *nirS* phylogeny. However, the primers proposed here for these clades performed well (Fig. 2). The clade-specific primers designed by Wei *et al*.^18^ for a selection of the known clades (Fig. 3) showed high specificity in general, but covered less than a third of the sequences in the clades they were meant to target (Fig. 2). In addition, they scored below the coverage of all other published primer sets for the proteobacterial clades of the two genes. Thus, the amplification bias shifted from low overall coverage and low specificity to high specificity with low coverage within each clade. Nevertheless, the *in silico* analyses performed in this study show primer options with higher coverage and reasonable specificity for various clades (Fig. 2).

### Primer performance against metagenomes

We retrieved a total of 648 *nirS* and 2,598 *nirK* metagenomic fragments, with an average abundance of 5.5 *nirS* and 4.1 *nirK* reads per gigabase sequenced in the metagenomes analyzed. This is orders of magnitude below what is detected in amplicon sequencing studies, underlining the need for PCR-based approaches to study nitrite reductase and other functional genes in natural environments. Matching all reads against the reference datasets^7^ resulted in fragmented alignments of partially overlapping regions. This compromises an unbiased estimation of coverage measures, but allows testing for specificity in a metagenomic dataset free of amplification biases.

We observed a distinct performance of primers across environments (Fig. 3). Weighted specificity, which accounts for the number of non-target clades detected by a given primer pair, ranged from 0.65 to 1, with some primers having no match to sequences in one environment while at the same time being highly specific for another. This is likely caused by compositional differences in the communities and indicates that the primers have biases towards certain lineages within the clades. Regardless, primer performance was mostly consistent across the three environments. Not all clades had identifiable representatives in all environments. For example, the *nirK* Firmicutes clade was not detected in any environment, whereas in other cases sequences matching clade-specific regions in the reference alignment were not retrieved in all environments, such as the epsilonproteobacterial clade from *nirS* in freshwater, thus preventing the evaluation of primers targeting these regions (Fig. 3). As expected, the highest specificity observed was for both *nirS* and *nirK* proteobacterial clades since their sequences have been used for primer design, and for clades with a smaller number of sequences due to their reduced diversity. The lowest specificity found was between the *nirK* clades of AniA and *Hyphomicrobium*, which comprise large and diverse clades. Primers with high coverage in the genome dataset (Fig. 2) generally showed low specificity in the metagenomic dataset (Fig. 3), confirming an expected trade-off between coverage and specificity. In consequence, the proteobacterial-specific primers proposed by Wei *et al*.^18^ showed high weighted specificity in all environments, but equal to that of the commonly used primers NirS1F and NirK5R^12^, R3cd^14^ and nirK1055R^20^.

## Discussion

Our results demonstrate the difficulty in designing primers that target the extant diversity of *nirS* and *nirK* genes, at least without resorting to prohibitively degenerate primers. We found no primer displaying both complete coverage and specificity at the same time, and the continuous increase in diversity with the addition of novel sequences plus the fact that no novel target regions exist for either gene, indicate that it may not be possible to obtain a single ideal universal primer pair for each of the *nir* genes. Primer design is complicated due to synonymous substitutions in the third codon position in protein-coding genes and there will likely be a non-conserved site every three nucleotides, even if there are strong selection forces acting towards the conservation of that particular amino acid residue. Thus, sequence identity will decrease as time from divergence increases, making the design of primers targeting ancient protein families like the nitrite reductases particularly difficult. Moreover, the phylogenetic history within these protein families is complex, shaped by vertical inheritance, horizontal gene transfer and duplication/divergence events^5^,^7^,^21^. However, they still show strong structural conservation, stressing the need to use alignments that are aware of protein structure to improve primer design.

For protein-coding genes in general, sequence divergence can be linked to a change in selection pressure that results in functional divergence. This can severely affect the ecological interpretation of results obtained, leading to an underestimation of the genetic potential for denitrification when certain clades are not targeted by the primer set, or possibly an overestimation if primers target homologous sequences that are not involved in denitrification. Moreover, nitrite reduction catalyzed by NirS and NirK occurs for different reasons in microorganisms and in different pathways in the nitrogen cycle^1^. Together, this advocates for clade-specific primers for ecological studies rather than using broad range primers. More precisely, primer design must be based on the delineation of ecologically relevant and evolutionarily coherent clades rather than simply defining lineages based on sequence identity alone and/or statistical significance. Defining evolutionarily coherent clades of *nir* genes and other functional genes will be especially beneficial for research examining the links between process rates and quantitative abundance measures (e.g. refs 22 and 23), allowing for rapid identification of clades of interest by quantitative PCR assays. Unfortunately, mutually exclusive clade-specific primer pairs with broad coverage are not available, and primers need to be selected by balancing high coverage with minimal overlap across clades (Figs 2 and  3) to avoid unrealistic abundance estimates. However, a main issue for quantitative studies is that primer pairs are seldom truly additive. Therefore, the total abundance of a specific functional guild will likely become heavily inflated when summing up the individual clades. This could explain the reported increase in detection power of summed clade-specific *nir* primers^18^ despite their low coverage (Fig. 2). There are also several problems associated with using clade-specific *nir* primers in sequenced-based community ecology studies. An inherent problem with the approach even when primers have high coverage and are truly clade specific is that it prevents characterization of the total community structure, thus hampering any inferences about differential lineage dominance both within and across environments. In addition, most primers display either low coverage, resulting in ecological patterns being overlooked, or low specificity. Non-specificity is not necessarily a problem if the objective is to characterize the structure of single clades, since spurious amplicons can be removed. This can be scaled up to high-coverage clade-specific primers when a single lineage is known to be dominant. However, using multiple primer pairs that are not mutually exclusive compromises any similarity measures between samples in OTU-based community analysis. This issue can be solved by mapping reads to robust reference phylogenies (e.g. ref. 24) and excluding spurious reads, although not without a large increase in workload.

An alternative approach for primer design is to use the observed performance differences between environments as a guide to develop primers specifically tailored to individual environments. This approach requires an initial exhaustive survey of the nucleotide diversity present in the target environment to describe the extant community composition, which could be done by sequencing the pooled product of multiple primer pairs. New primers would be designed from that initial survey targeting the relevant groups found, minimizing primer biases and improving efforts to quantify the abundance and diversity of targeted functional groups in the environment of interest. With the repeated sampling of similar environments in time and space, a well-defined collection of primers could be identified for a particular environmental type (e.g. wastewater, marine sediment, etc.), though this of course depends on the complexity of each environment. This approach is similar in concept to that outlined by Kushwaha *et al*.^25^, in which metagenomics datasets are used to design molecular probes targeting specific sets of functional genes for use in sequence-capture techniques. A PCR-based approach using primers designed in this manner would be more sensitive in detecting functional genes of low relative abundance compared to probe-capture techniques, and the primers could also be used for quantitative analyses.

The growing number of sequences deposited in databases makes the development and continuous update of primer sets a bottleneck in microbial ecology of denitrifiers, other nitrite reducers and functional groups in general, as estimates of coverage and specificity of primer sets require comprehensive phylogenetic analyses of highly divergent sequences and demonstration of their performance using environmental samples. Traditionally, primer evaluation has included the demonstration of successful amplification in a number of strains known to harbor the gene and sequencing of a few environmental samples^14^,^18^,^19^. But this is an unrealistic and biased representation of microbial diversity. We suggest this should be complemented by testing (i) coverage and phylogenetic range using full-length sequences from complete genomes, and (ii) specificity and environmental range with available metagenomes. The framework presented here can be used as a guideline to facilitate comparison of primer performance, allowing for the integration of results obtained in different studies into one single ecological context. This should be done on existing primer sets even if we cannot redesign or develop new primers since it would clarify the limitations and biases of the primers used, as exemplified in our analyses of *nir* genes.

We conclude that an exhaustive description of *nir* gene diversity and abundance in the environment will require multiple complementary primer sets, despite the issues discussed here. This does not represent a larger effort than the implementation of novel methods targeting microbial communities like synthetic long reads^26^ or single-molecule real time sequencing^27^, especially since novel methods require new developments and rigorous testing. The use of multiple primer pairs, by contrast, relies on familiar methodologies and concepts for PCR amplification. The phylogenetic evaluation of *nir* primer pairs in our study can be used as a reference along with their environmental specificity as a coarse guide for primer selection and future primer design. Furthermore, researchers can avoid the potential pitfalls of *nir* gene analyses by implicit incorporation of currently known biases and theoretical contingencies into primer selection and experimental design, combined with the explicit acknowledgement of such issues in ecological interpretations of results.

## Methods

### Primer design and primer data set

To design novel broad spectrum and cluster-specific *nirS* and *nirK* primers, we used the most up to date, comprehensive and robust phylogenies published by Graf *et al*.^7^, and aligned the 454 and 110 full-length *nirK* and *nirS* sequences from complete genomes using the automatic structure guided alignment implemented in *t-coffee expresso*^28^. For primer design we used HYDEN^29^ and DegePrime^30^, which resulted in a total of 77 *nirS* and 79 *nirK* primer candidates (Table S1). To this dataset, we added the most commonly used primers for both genes (Fig. 2) and the clade-specific primers recently developed by Wei *et al*.^18^. Since fungal *nirK* show high similarity to bacterial sequences, we included primer sets available for the eukaryotic clade^31^,^32^. Thaumarchaeal sequences display extensive divergence from the rest of the *nirK* sequences, and primer evaluation was excluded from the present study since it has been discussed elsewhere^33^.

### Phylogenetic coverage and specificity

To estimate phylogenetic coverage and specificity of the primers, we evaluated all primers *in silico* against the phylogenies using ARB^34^ (Tables S2 and S3). A hit was considered when both primers in a pair matched the same sequence with a maximum of three mismatches. Three aspects of performance were measured: *coverage* as the proportion of sequences hit in the targeted clade, the number of *non-target clades* hit, and *weighted specificity* defined as the proportion of non-target sequences hit to the total number of non-target sequences, weighted by the proportion of non-target clades hit to the total number of non-target clades.

### Performance with environmental metagenomes

While testing primers against full-length sequences from complete genomes allows for an accurate evaluation against a phylogenetically comprehensive dataset, it is also necessary to estimate their performance with environmental datasets. For this, we retrieved fragments homologous to *nirK* and *nirS* from 47 metagenomes publicly available in IMG/M using *blastp* and retrieving their respective nucleotide sequences^35^ (Table S4). We searched metagenomes from three different types of environments (soil, industrial wastewater and freshwater). These environments were chosen as they represent a subset of habitats with contrasting compositions of communities of denitrifiers, are the subject of intense study on denitrification from different research areas, and because their metagenomes allow for comparison regarding the sequencing platform used and sequencing depth. The fragments were translated with HMMFrame^36^, aligned against the reference alignments and mapped against the reference phylogenies^7^ using Evolutionary Placement in *RAxML*^37^. This produced a fragmented alignment with numerous partially overlapping regions, allowing only the performance evaluation of individual primers instead of by primer pairs. Since this prevents the evaluation of coverage, performance in metagenomes focuses on weighted specificity. In this case, total sequences were counted separately as the totals present in each of the three environments analyzed.

## Additional Information

**How to cite this article**: Bonilla-Rosso, G. *et al*. Design and evaluation of primers targeting genes encoding NO-forming nitrite reductases: implications for ecological inference of denitrifying communities. *Sci. Rep.* **6**, 39208; doi: 10.1038/srep39208 (2016).

**Publisher’s note:** Springer Nature remains neutral with regard to jurisdictional claims in published maps and institutional affiliations.

## Supplementary Material

Supplementary Material
