# Supplementary material for: Design and evaluation of primers targeting genes encoding NO-forming nitrite reductases: implications for ecological inference of denitrifying communities
Source: Sci Rep. 2016 Dec 14;6:39208. doi: 10.1038/srep39208 (PMC5155301; doi:10.1038/srep39208)
Supplement: Supplementary Material [file srep39208-s1.pdf]

Supplementary material

Design and evaluation of primers targeting genes encoding NO-forming nitrite reductases: implications for ecological inference of denitrifying communities

Germán Bonilla-Rosso, Lea Wittorf, Christopher M. Jones and Sara Hallin*

Content:

Table S1. Sequences of all *nirS* and *nirK* primers used, grouped by targeted clade.

Table S2. Coverage and specificity of all *nirS* primer pairs evaluated against full length sequences from complete genomes. Weighted specificity is a measure of the proportion of non-target sequences hit to the total number of non-target sequences, divided by the proportion of non-target clades hit to the total number of non-target clades.

Table S3. Coverage and specificity of *nirK* primer pairs evaluated against full length sequences from complete genomes. Weighted specificity is a measure of the proportion of non-target sequences hit to the total number of non-target sequences, divided by the proportion of non-target clades hit to the total number of non-target clades

Table S4. Metagenomes from where *nirS* and *nirK* sequences were retrieved. They are grouped by environment and ecosystem categories as reported in IMG.

Table S1. Sequences of all *nirS* and *nirK* primers used, grouped by targeted clade.

Gene	Clade	Name	Sequence (5'-3')	Reference
nirK	Proteobacteria (all)	Cunir3F	CGTCTAYCAYTGCGCNCC	1
		F1aCu	ATCATGGTSCTGCCGCG	2
		R3Cu	GCCTCGATCAGRTTGTGGTT	2
		nirK1F	GGMATGGTKCCSTGGCA	3
		nirK3R	GAACTTGCCGGTNGYCCAGAC	3
		nirK5R	GCCTCGATCAGRTTRTGG	3
		nirK517F	TTYGTSTAYCACTGCGCVCC	4
		nirK1055R	GCYTCGATCAGRTTRTGGTT	4
		nirKC1F	ATGGCGCCATCATGGTNYTNCC	5
		nirKC1R	TCGAAGGCCTCGATNARRTTRTG	5
		nirKP1_1R	GCRTANANNCCNGGYTG	This study
		nirKP1_2R	TNRTNGGNCANKGNGA	This study
		nirKP1_3R	TGACATAGGCGTAGATGC	This study
		nirKP1_4R	CCCAGACATAATCGCC	This study
		nirKP1_1F	AAYATNGAYTTYCAYTC	This study
		nirKP1_2F	AANRTNGAYTTNCAYKC	This study
		nirKP1_3F	AANRTNGAYYTNCAYKC	This study
		nirKP1_4F	ANRTNGAYTTNCAYKC	This study
		nirKP1_5F	ANRTNGAYYTNCAYKC	This study
		nirKP1_6F	GYNANYGGNATGWWCGG	This study
		nirKP1_7F	CAYRTNGYNAAYGGNATG	This study
		nirKP1_8F	CAYATNGCNAAYGGNATG	This study
		nirKP1_5R	TYNCCNTGNCCNCCNAT	This study
		nirKP1_6R	TGNCCNCCNAYNANRTG	This study
		nirKP1_7R	GCYTNRATCARRTTRTG	This study
		nirKP1_9F	YNTNTAYCAYTGYGCNCC	This study
		nirKP1_10F	TNTAYCAYTGYGCNCC	This study
		nirKP1_11F	YNTNTAYCAYTGYGC	This study
		nirKP1_12F	YNYNTAYCAYTGYGC	This study
		nirKP1_13F	TTYGTCTAYCACTGCGC	This study
		nirKP1_8R	TCNTCRCGCGGCAGNACCAT	This study
	AniA	nirKC2F	TGCACATCGCCAACGGNATGTWYGG	5
		nirKC2R	GGCGCGGAAGATGSHRTGRTCNAC	5
		nirKA2_1F	TYGCCAACGGNATGTAYGG	This study
		nirKA2_1R	RRCCRTACATNCCGTTGGC	This study
		nirKA2_2F	TGCARGGNGAYTTYTAYAC	This study
		nirKA2_2R	GCCARYGGMGGRAACRC	This study
		nirKA2_3F	TGCARGGYGANTTYTAYAC	This study
		nirKA2_3R	GCCARYGGMGGRAACRCC	This study
		nirKA2_4F	GCNAAYGGNATGTAYGG	This study
		nirKA2_5F	TGCARGGNGAYTTYTAYAC	This study
		nirKA2_4R	CGGAARATNGARTGRTC	This study
		nirKA2_5R	RTCRAARATYTCRCCRAT	This study
		nirKA2_6R	AARATMGAGTGRTCNAC	This study
		nirKA2_7R	TCRAARATYTCRCCRATNA	This study
		nirKA2_8R	CYTTRTTRAANGYRCGGAA	This study
	Haloarchaea	nirKA4_1R	TGTASAGYTCGTKYTGNCC	This study
		nirKA4_1F	TCTACCACTGYGCSGTSCC	This study
		nirKA4_2R	TCGTKCTGNCCSARGTAGA	This study
		nirKA4_2F	TCTACCACTGYGCSRTSCC	This study
		nirKA4_3F	TCTACCACTGYGCSGTSCCG	This study
		nirKA4_3R	TCGTKYTGSCCGARGTAGA	This study
	Actinobacteria	nirKC3F	CATCGGCAACGGCATGYAYGGNGC	5
		nirKC3R*	CGACCATGGCGTGGSWNACRAANGG	5
		nirKA6_1R	CTCSACRAANCCGCCYTGS	This study
		nirKA6_2R	TCSACRAANCCGCCYTGSG	This study
		nirKA6_1F	TCTGGMTSTAYCACTGCKC	This study
		nirKA6_3R	TCSACGAANCCGCCCTGSG	This study
	Hyphomicrobia	NirK-R	TTGCATGACGTAGAACTCGC	6
		nirKH3_1F	TKCARATCAMYCTSRTCAA	This study
		nirKH3_1R	CCTGCATSACRTAGAANTC	This study
		nirKH3_2F	GAGTTCTAYGTSATGCAGS	This study
		nirKH3_2R	TCGAANATYTCNCCGATS	This study
		nirKH3_3R	TCGCCGATSACRTGGAA	This study
	Nitrosomonas	nirKC4F	TACGGTGTGATCATCRTSGATCC	5
		nirKC4R	GCATCACGCATGGAATGATYSAC	5
		nirKN7_1R	TCCCAGATNCCRGCRATCG	This study
		nirKN7_1F	CAYATYGCNCGCGGTATG	This study
		nirKN7_2R	TCCCAGATNCCRGCRATCG	This study
		nirKN7_3R	RTCATGCACSGGRTCRTA	This study
		nirKN7_4R	RTCATGNACNGGRTCRTA	This study
		nirKN7_2F	ATGNTYCAGCAYATYGCNCG	This study
		nirKN7_3F	TYCAGCAYATYGCNCGNGG	This study
	Eukaryotes	EunirK-F1	GGBAAYCCICAYAAYATCGA	7
		EunirK-R1	GGICCIGCRTTSCCRAAGAA	7
		nirKfF	TACGGGCTCATGTAYGTNSARCC	8
		nirKfR	AGGAATCCCACASCNCCYTTNTC	8
	Firmicutes	GnirK2F	GGKGTVTTTATGTACCATTGC	9
		GnirK2R	SCCGCTYGCCGGAAGCATCAC	9

nirS	Proteobacteria (all)	cd3aF	GTSAACGTSAAGGARACSGG	10
		F1bcd	TAYCACCCSGARCCGCG	11
		NirS1F	CCTAYTGGCCGCCRCART	3
		NirS3Fa	TAYTGGCCSCCRCARTWC	11
		NirS3Fb	YNTAYTGGCCSCCRCARTWC	This study
		NirS3Fg	YNTAYTGGCCGCCRCARTWC	This study
		NirS4R	TTCGGRTGSGTCTTGAYGAA	3
		NirS6R	CGTTGAACTTRCCGGT	3
		R3cd	GASTTCGGRTGSGTCTTGA	11
		R4bcd	CGTTGAAYTTRCCGGTSGG	11
		nirSC1F	ATCGTCAACGTCAARGARACVGG	5
		nirSC1R	TTCGGGTGCGTCTTSAYGAASAG	5
		nirSP1_1F	YGCTGCGCAARGGNGCNACSGG	This study
		nirSP1_2F	GCTGCGCAARGGNGCNACSGG	This study
		nirSP1_3F	AAYGTNAARGARACCGGC	This study
		nirSP1_4F	YTAYTGGCCNCCGCANTW	This study
		nirSP1_5F	TAYTGGCCSCCRCARTWC	This study
		nirSP1_6F	YNTAYTGGCCSCCRCARTWC	This study
		nirSP1_7F	YNTAYTGGCCGCCRCARTWC	This study
		nirSP1_8F	GGNTAYGCNGTTCAYATY	This study
		nirSP1_9F	GGYATGCCSRACTGGGNNA	This study
		nirSP1_10F	NAAYGTNAARGARACCGG	This study
		nirSP1_11F	TSAACGYNAAGGARAYSGG	This study
		nirSP1_12F	TNAASGYNAAGGARACSGG	This study
		nirSP1_13F	TAYCACCCNGARCCNCG	This study
		nirSP1_14F	TAYCAYCCNGARCCNCG	This study
		nirSP1_15F	TWYCAYCCNGARCCNCG	This study
		nirSP1_16F	TAYCACCCNGARCCNCG	This study
		nirSP1_1R	NGGRTGGGTCTTSANRAA	This study
		nirSP1_2R	ACRTTGAANTKRCCGGTNGG	This study
		nirSP1_3R	RTTGAANTKRCCGGTNGG	This study
		nirSP1_4R	TNGGRTGNGTYTTSAGRAA	This study
		nirSP1_5R	GTTGGCRCCNCGNCCNGG	This study
		nirSP1_6R	GYTGGCGCCRCGNCCNGG	This study
		nirSP1_7R	GTTGGCGCCNCGNCCNGGRTG	This study
		nirSP1_17F	GYTGYGCNGGYTGYCAYG	This study
		nirSP1_18F	AAYGTNAARGARACCGGC	This study
		nirSP1_19F	TSAACGYNAAGGARACSGG	This study
		nirSP1_20F	TAYTGGCCGCCNCARTWC	This study
		nirSP1_8R	GTTGAANTTNCCSGTNGG	This study
		nirSP1_9R	GAACCASAYYTCGTCRCC	This study
		nirSP1_10R	GGRTGSGTCTTSANGAAC	This study
		nirSP1_21F	TAYTGGCCRCCNCARTNC	This study
		nirSP1_11R	GGRTGSSTCTTSANGAA	This study
		nirSP1_22F	ACCGGNTAYGCCGTNCA	This study
		nirSP1_23F	AAYRYSAAGGARACSGG	This study
	Epsilonproteobacteria	nirSC3F	TTCGCCCTGAARGAYGGNGG	5
		nirSC3R	AGGTGCCCACGAANARNCCNCC	5
		nirSE3_1F	AGARTGYCARGGNTGTCAYGG	This study
		nirSE3_2F	GTYTCWCCMGAYGGWAAATA	This study
		nirSE3_3F	ACWCCWTAYGSWCCNTAT	This study
		nirSE3_4F	GGNGATATTCCNAAYATY	This study
		nirSE3_1R	GTACCNACAAANAAWCCRCCAC	This study
		nirSE3_2R	GAACCTTGWCCTGGGTGYGG	This study
		nirSE3_3R	TAYTTRACRAAYTTWCCWG	This study
		nirSE3_4R	NKTCCACTCRGAAAYCAT	This study
		nirSE3_5R	CCRACWKTAATRATTCTR	This study
	Thermophilic	nirST2_1F	TGGGNTGCTACTGGCCNC	This study
		nirST2_2F	AARGAGNCSGGCCAGRYCTGGCT	This study
		nirST2_3F	GNTGNTAYTGGCCNCC	This study
		nirST2_4F	GGAATGCCCGAYTGGGGNM	This study
		nirST2_5F	AGGTKGCCATCATYGAYGGNGA	This study
		nirST2_6F	GRYCTGGCTNGTNGACTAC	This study
		nirST2_1R	ACGTTGAACTTNCCYGTNGG	This study
		nirST2_2R	TKGCMGCMACRAGGAAGTA	This study
		nirST2_3R	GGTGGCCASTASCABCCNA	This study
		nirST2_4R	GTANACRTTGAACTTNCCYGT	This study
		nirST2_5R	CYGTNGGNGTKCNCACCCA	This study
		nirST2_6R	GGRTGGGTYTTGATRAAGAG	This study
	Archaea	nirSA4_1F	TCNAAGATAGATYTSTAY	This study
		nirSA4_2F	TNCAARATMGAYCTGTWC	This study
		nirSA4_3F	TMCAARATMGAYCTGTWC	This study
		nirSA4_4F	CTNTNCAARATMGAYCTGTWC	This study
		nirSA4_5F	CTNTNCAARATMGAYCTG	This study
		nirSA4_6F	ATCNTCGACGCNGANRCS	This study
		nirSA4_1R	CCAMGTCCATNGTCTGNA	This study
		nirSA4_2R	RNAGWACAKNCCSCCGCC	This study

*Primer sequence has been revised from published sequence by one base

Table S2. Coverage and specificity of all *nirS* primer pairs evaluated against full length sequences from complete genomes.Weighted specificity is a measure of the proportion of non-target sequences hit to the total number of of non-target sequences, divided by the proportion of non-target clades hit to the total number of non-target clades.

Clade	Forward Primer	Reverse Primer	Mismatches (min 0, max X)	Coverage per clade and total						Weighted Specificity	Non-target clades
				Proteobacteria	Thermophilic	Epsilon-proteobacteria	Archaea	Others	Total		
Proteobacteria	cd3AF	R3cd	2	0,76	0,10	0,00	0,00	0,00	0,57	0,99	1
			3	0,84	0,30	0,00	0,00	0,22	0,67	0,91	2
	cd3aF	R4bcd	2	0,83	0,30	0,00	0,00	0,33	0,67	0,89	2
			3	0,95	0,60	0,00	0,00	0,44	0,80	0,82	2
	cd3aF	NirS6R	2	0,82	0,30	0,00	0,00	0,33	0,66	0,89	2
			3	0,95	0,60	0,00	0,00	0,44	0,80	0,82	2
	cd3aF	NirS4R	2	0,73	0,30	0,00	0,00	0,22	0,59	0,91	2
			3	0,88	0,50	0,00	0,00	0,22	0,72	0,88	2
	NirS1F	R3cd	2	0,78	0,00	0,00	0,00	0,11	0,69	0,99	1
			3	1,00	0,00	0,00	0,00	0,67	0,76	0,95	1
	NirS1F	NirS6R	2	0,84	0,00	0,00	0,00	0,33	0,65	0,97	1
			3	0,98	0,00	0,00	0,00	0,67	0,78	0,95	1
	NirS1F	NirS4R	2	0,76	0,00	0,00	0,00	0,11	0,57	0,99	1
			3	0,90	0,00	0,00	0,00	0,11	0,68	0,99	1
	F1bcd	R3cd	2	0,77	0,00	0,00	0,00	0,11	0,58	0,99	1
			3	0,85	0,00	0,00	0,00	0,33	0,66	0,97	1
	F1bcd	NirS6R	2	0,84	0,00	0,00	0,00	0,22	0,65	0,98	1
			3	0,98	0,00	0,00	0,00	0,33	0,75	0,97	1
	F1bcd	R4bcd	2	0,85	0,00	0,00	0,00	0,22	0,65	0,98	1
			3	0,98	0,10	0,00	0,00	0,33	0,76	0,93	2
	F1bcd	NirS4R	2	0,74	0,00	0,00	0,00	0,22	0,57	0,98	1
			3	0,90	0,00	0,00	0,00	0,33	0,70	0,97	1
	nirSC1F	nirSC1R	2	0,33	0,00	0,00	0,00	0,22	0,26	0,98	1
			3	0,60	0,20	0,00	0,00	0,22	0,48	0,93	2
	nirSP1_21F	nirSP1_11R	2	1,00	0,00	0,00	0,00	0,33	0,77	0,97	1
			3	1,00	0,10	0,00	0,00	0,67	0,81	0,88	2
	niRSP1_22F	nirSP1_11R	2	1,00	0,40	0,00	0,00	0,33	0,81	0,88	2
			3	1,00	0,80	0,00	0,00	0,67	0,87	0,75	2
	nirSP1_23F	nirSP1_11R	2	0,98	0,60	0,00	0,00	0,33	0,81	0,84	2
			3	1,00	1,00	0,00	0,00	0,56	0,88	0,73	2
	F1bcd	nirSP1_11R	2	0,96	0,00	0,00	0,00	0,22	0,74	0,98	1
			3	1,00	0,10	0,20	0,00	0,33	0,79	0,87	3
	NirS1F	nirSP1_11R	2	0,96	0,00	0,00	0,00	0,11	0,73	0,99	1
			3	1,00	0,00	0,00	0,00	0,44	0,78	0,96	1
	nirSP1_17F	nirSP1_5R	2	1,00	0,70	0,00	0,00	1,00	0,89	0,71	2
			3	1,00	0,90	0,00	0,00	1,00	0,91	0,68	2
	nirSP1_17F	nirSP1_6R	2	1,00	0,40	0,00	0,00	0,44	0,82	0,86	2
			3	1,00	0,90	0,00	0,00	0,89	0,90	0,70	2
	nirSP1_8F	nirSP1_1R	2	0,96	0,40	0,00	0,00	0,33	0,78	0,88	2
			3	1,00	0,60	0,00	0,00	0,56	0,85	0,80	2
	nirSP1_8F	nirSP1_5R	2	1,00	0,30	0,00	0,00	0,44	0,81	0,88	2
			3	1,00	0,50	0,00	0,25	1,00	0,88	0,60	3
	nirSP1_8F	nirSP1_6R	2	1,00	0,20	0,00	0,00	0,44	0,80	0,89	2
			3	1,00	0,50	0,00	0,00	0,89	0,86	0,77	2
	nirSP1_10F	NirS6R	2	0,85	0,20	0,00	0,00	0,44	0,69	0,89	2
			3	0,98	0,90	0,00	0,00	0,89	0,88	0,70	2
	nirSP1_10F	R4bcd	2	0,87	0,20	0,00	0,00	0,44	0,70	0,89	2
			3	0,98	0,90	0,00	0,00	0,89	0,88	0,70	2
	nirSP1_10F	nirSP1_1R	2	0,94	0,40	0,00	0,00	0,33	0,76	0,88	2
			3	1,00	1,00	0,00	0,00	0,44	0,87	0,75	2
	nirSP1_10F	nirSP1_2R	2	0,96	0,40	0,00	0,00	0,44	0,79	0,86	2
			3	1,00	1,00	0,00	0,00	0,89	0,91	0,68	2
	nirSP1_10F	nirSP1_3R	2	0,98	0,40	0,00	0,00	0,44	0,80	0,86	2
			3	1,00	1,00	0,00	0,00	0,89	0,91	0,68	2
	nirSP1_10F	nirSP1_8R	2	0,98	0,40	0,00	0,00	0,44	0,80	0,86	2
			3	1,00	1,00	0,00	0,00	0,89	0,91	0,68	2
	nirSP1_15F	NirS6R	2	0,88	0,10	0,00	0,00	0,33	0,69	0,93	2
			3	0,98	0,60	0,00	0,00	0,78	0,85	0,77	2
	nirSP1_15F	R4bcd	2	0,89	0,10	0,00	0,00	0,33	0,70	0,93	2
			3	0,98	0,60	0,00	0,00	0,78	0,85	0,77	2
	nirSP1_15F	nirSP1_1R	2	0,96	0,10	0,00	0,00	0,33	0,75	0,93	2
			3	1,00	0,60	0,00	0,00	0,56	0,85	0,80	2
	nirSP1_15F	nirSP1_4R	2	1,00	0,10	0,00	0,00	0,33	0,78	0,93	2
			3	1,00	0,60	0,00	0,00	0,33	0,83	0,84	2
	nirSP1_15F	nirSP1_10R	2	0,96	0,00	0,00	0,00	0,33	0,75	0,97	1
			3	1,00	0,60	0,00	0,00	0,33	0,83	0,84	2
	nirSP1_15F	nirSP1_2R	2	0,99	0,10	0,00	0,00	0,33	0,77	0,93	2
			3	1,00	0,60	0,00	0,00	0,78	0,86	0,77	2
	nirSP1_15F	nirSP1_3R	2	1,00	0,10	0,00	0,00	0,33	0,78	0,93	2
			3	1,00	0,60	0,00	0,00	0,78	0,86	0,77	2
	nirSP1_15F	nirSP1_8R	2	1,00	0,10	0,00	0,00	0,33	0,78	0,93	2
			3	1,00	0,60	0,00	0,00	0,78	0,86	0,77	2
	nirSP1_20F	nirSP1_1R	2	0,95	0,00	0,00	0,00	0,33	0,74	0,97	1
			3	1,00	0,00	0,00	0,00	0,56	0,79	0,96	1
	nirSP1_20F	nirSP1_4R	2	0,99	0,00	0,00	0,00	0,33	0,76	0,97	1
			3	1,00	0,00	0,00	0,00	0,33	0,77	0,97	1
	nirSP1_20F	nirSP1_9R	2	0,95	0,00	0,00	0,00	0,00	0,71	1,00	0
			3	0,99	0,00	0,00	0,00	0,33	0,76	0,97	1
	nirSP1_20F	nirSP1_10R	2	0,95	0,00	0,00	0,00	0,33	0,74	0,97	1
			3	1,00	0,00	0,00	0,00	0,33	0,77	0,97	1
	nirSP1_20F	nirSP1_8R	2	0,99	0,00	0,00	0,00	0,89	0,81	0,93	1
			3	1,00	0,00	0,00	0,00	1,00	0,83	0,92	1
	nirSP1_4F	nirSP1_1R	2	0,96	0,00	0,00	0,00	0,33	0,75	0,97	1
			3	1,00	0,20	0,00	0,00	0,56	0,81	0,88	2
	nirSP1_4F	nirSP1_4R	2	1,00	0,00	0,00	0,00	0,33	0,77	0,97	1
			3	1,00	0,20	0,00	0,00	0,33	0,79	0,91	2
	nirSP1_4F	nirSP1_9R	2	0,96	0,00	0,00	0,00	0,00	0,72	1,00	0
			3	0,99	0,00	0,00	0,00	0,33	0,76	0,97	1
	nirSP1_4F	nirSP1_10R	2	0,96	0,00	0,00	0,00	0,33	0,75	0,97	1
			3	1,00	0,20	0,00	0,00	0,33	0,79	0,91	2
	nirSP1_4F	nirSP1_8R	2	1,00	0,00	0,00	0,00	1,00	0,83	0,92	1
			3	1,00	0,20	0,00	0,00	1,00	0,85	0,80	2
	nirSP1_9F	nirSP1_6R	2	0,80	0,50	0,00	0,00	0,33	0,67	0,86	2
			3	0,83	0,90	0,00	0,00	0,44	0,74	0,77	2
	nirSP1_9F	nirSP1_1R	2	0,77	0,80	0,00	0,00	0,33	0,67	0,80	2
			3	0,83	1,00	0,00	0,00	0,44	0,75	0,75	2
	nirSP1_9F	nirSP1_4R	2	0,80	0,80	0,00	0,00	0,33	0,70	0,80	2
			3	0,83	1,00	0,00	0,00	0,33	0,74	0,77	2
	nirSP1_9F	nirSP1_9R	2	0,78	0,00	0,00	0,00	0,11	0,59	0,99	1
			3	0,83	0,10	0,00	0,00	0,11	0,64	0,96	2
	nirSP1_9F	nirSP1_10R	2	0,77	0,60	0,00	0,00	0,33	0,65	0,84	2
			3	0,83	0,90	0,00	0,00	0,33	0,73	0,79	2
	nirSP1_13F	nirSP1_9R	2	0,96	0,00	0,00	0,00	0,33	0,75	0,97	1
			3	1,00	0,20	0,00	0,00	0,33	0,79	0,91	2
	nirSP1_13F	nirSP1_4R	2	1,00	0,00	0,00	0,00	0,33	0,77	0,97	1
			3	1,00	0,20	0,00	0,00	0,33	0,79	0,91	2
Thermophilic	nirST2_1F	nirST2_5R	2	0,00	0,80	0,00	0,00	0,00	0,07	1,00	0
			3	0,00	0,80	0,00	0,00	0,00	0,07	1,00	0
	nirST2_1F	nirST2_6R	2	0,02	0,70	0,00	0,00	0,33	0,11	0,98	2
			3	0,21	0,80	0,00	0,00	0,33	0,25	0,90	2
	nirST2_6F	nirST2_5R	2	0,00	0,80	0,00	0,00	0,00	0,07	1,00	0
			3	0,01	0,90	0,00	0,00	0,11	0,10	0,99	2
	nirST2_6F	nirST2_6R	2	0,10	0,80	0,00	0,00	0,22	0,16	0,95	2
			3	0,50	0,90	0,00	0,00	0,33	0,48	0,78	2
	nirSP1_10F	nirST2_5R	2	0,00	0,30	0,00	0,00	0,00	0,03	1,00	0
			3	0,05	1,00	0,00	0,00	0,00	0,13	0,99	1
	nirSP1_10F	nirST2_6R	2	0,71	0,30	0,00	0,00	0,33	0,58	0,70	2
			3	0,87	1,00	0,00	0,00	0,33	0,76	0,63	2
Epsilonproteobacteria	nirSC3F	nirSE3_1R	2	0,00	0,00	0,40	0,00	0,00	0,02	1,00	0
			3	0,00	0,00	0,60	0,00	0,00	0,03	1,00	0
	nirSC3F	nirSE3_4R	2	0,00	0,00	0,40	0,00	0,00	0,02	1,00	0
			3	0,00	0,00	0,40	0,00	0,00	0,02	1,00	0
	nirSC3F	nirSE3_3R	2	0,00	0,00	0,40	0,00	0,00	0,02	1,00	0
			3	0,00	0,00	0,40	0,00	0,00	0,02	1,00	0
	nirSE3_2F	nirSE3_1R	2	0,00	0,00	0,80	0,00	0,00	0,04	1,00	0
			3	0,00	0,00	1,00	0,00	0,00	0,05	1,00	0
	nirSE3_2F	nirSE3_4R	2	0,00	0,00	0,80	0,00	0,00	0,04	1,00	0
			3	0,00	0,00	0,80	0,00	0,00	0,04	1,00	0
	nirSE3_2F	nirSE3_3R	2	0,00	0,00	0,80	0,00	0,00	0,04	1,00	0
			3	0,00	0,00	0,80	0,00	0,00	0,04	1,00	0
	nirSE3_3F	nirSE3_1R	2	0,00	0,00	0,80	0,00	0,00	0,04	1,00	0
			3	0,00	0,00	1,00	0,00	0,00	0,05	1,00	0
	nirSE3_3F	nirSE3_4R	2	0,00	0,00	0,80	0,00	0,00	0,04	1,00	0
			3	0,00	0,00	0,80	0,00	0,00	0,04	1,00	0
	nirSE3_3F	nirSE3_3R	2	0,00	0,00	0,80	0,00	0,00	0,04	1,00	0
			3	0,00	0,00	0,80	0,00	0,00	0,04	1,00	0
	nirSE3_4F	nirSE3_1R	2	0,00	0,00	0,80	0,00	0,00	0,04	1,00	0
			3	0,00	0,00	1,00	0,00	0,00	0,05	1,00	0
	nirSE3_4F	nirSE3_4R	2	0,00	0,00	0,80	0,00	0,00	0,04	1,00	0
			3	0,00	0,00	0,80	0,00	0,00	0,04	1,00	0
	nirSE3_4F	nirSE3_3R	2	0,00	0,00	0,80	0,00	0,00	0,04	1,00	0
			3	0,00	0,00	0,80	0,00	0,00	0,04	1,00	0
	nirSC3F	nirSC3R	2	0,00	0,00	0,00	0,00	0,00	0,00	1,00	0
			3	0,00	0,00	0,20	0,00	0,00	0,01	1,00	0
Archaea	nirSA4_1F	nirSA4_1R	2	0,00	0,00	0,00	1,00	0,00	0,04	1,00	0
			3	0,00	0,00	0,20	1,00	0,00	0,05	1,00	1
	nirSA4_1F	nirSA4_2R	2	0,00	0,00	0,00	1,00	0,00	0,04	1,00	0
			3	0,00	0,00	0,20	1,00	0,00	0,05	1,00	1
	nirSA4_3F	nirSA4_1R	2	0,00	0,00	0,00	1,00	0,00	0,04	1,00	0
			3	0,00	0,00	0,00	1,00	0,00	0,04	1,00	0
	nirSA4_2F	nirSA4_1R	2	0,00	0,00	0,00	1,00	0,00	0,04	1,00	0
			3	0,00	0,00	0,20	1,00	0,00	0,05	1,00	1
	nirSA4_4F	nirSA4_1R	2	0,00	0,00	0,00	0,00	0,00	0,00	1,00	0
			3	0,00	0,00	0,00	1,00	0,11	0,05	1,00	1
	nirSA4_4F	nirSA4_2R	2	0,00	0,00	0,00	1,00	0,00	0,04	1,00	0
			3	0,00	0,00	0,00	1,00	0,11	0,05	1,00	1

Table S3. Coverage and specificity of all *nirK* primer pairs evaluated against full length sequences from complete genomes. Weighted specificity is a measure of the proportion of non-target sequences hit to the total number of non-target sequences, divided by the proportion of non-target clades hit to the total number of non-target clades.

Clade	Forward Primer	Reverse Primer	Mismatches (min 0, max X)	Coverage per clade and total													Weighted Specificity	Non-target clades
				Proteobacteria	AniA	Haloarchaea	Eukaryotes	Actinobacteria	Burkholderia	Thaum-archaeota	Thioalkali-vibrio	Nitrosomonas	Firmicutes	Hypho-microbia	Others	Total		
all	nirKP1_1F	nirKP1_1R	2	0,93	0,27	0,09	0,32	0,01	0,00	0,14	0,00	0,08	0,00	0,05	0,00	0,34	-	-
			3	1,00	0,77	0,91	0,75	0,38	0,00	1,00	0,00	0,54	0,44	0,24	0,29	0,67	-	-
	nirKP1_2F	nirKP1_1R	2	1,00	0,28	0,18	0,39	0,13	0,00	1,00	0,00	0,08	0,00	0,33	0,07	0,42	-	-
			3	1,00	0,88	1,00	0,75	0,87	0,80	1,00	0,75	0,77	0,69	1,00	1,00	0,90	-	-
	nirKP1_3F	nirKP1_1R	2	1,00	0,28	0,18	0,39	0,13	0,00	1,00	0,25	0,15	0,00	0,48	0,21	0,44	-	-
			3	1,00	0,88	1,00	0,75	0,90	1,00	1,00	0,75	0,77	0,69	1,00	1,00	0,91	-	-
	nirKP1_4F	nirKP1_1R	2	1,00	0,28	0,18	0,39	0,15	0,00	1,00	0,00	0,23	0,00	0,48	0,29	0,44	-	-
			3	1,00	0,88	1,00	0,75	0,90	1,00	1,00	1,00	0,77	0,69	1,00	1,00	0,91	-	-
	nirKP1_5F	nirKP1_1R	2	1,00	0,28	0,18	0,39	0,16	0,00	1,00	0,25	0,23	0,00	0,48	0,29	0,45	-	-
			3	1,00	0,88	1,00	0,75	0,90	1,00	1,00	1,00	0,77	0,69	1,00	1,00	0,91	-	-
	nirKP1_1F	nirKP1_2R	2	0,93	0,65	0,91	0,93	0,03	0,00	0,14	0,00	0,08	0,50	0,05	0,14	0,52	-	-
			3	1,00	0,89	0,91	1,00	0,41	0,00	1,00	0,00	0,62	0,75	0,24	0,29	0,74	-	-
	nirKP1_1F	nirKP1_3R	2	0,43	0,00	0,00	0,00	0,00	0,00	0,00	0,00	0,00	0,00	0,00	0,00	0,11	-	-
			3	0,63	0,00	0,00	0,00	0,00	0,00	0,00	0,00	0,00	0,00	0,00	0,00	0,16	-	-
	nirKP1_1F	nirKP1_4R	2	0,58	0,01	0,00	0,00	0,00	0,00	0,00	0,00	0,00	0,00	0,00	0,00	0,15	-	-
			3	0,81	0,01	0,00	0,00	0,00	0,00	0,00	0,00	0,00	0,00	0,00	0,00	0,21	-	-
	nirKP1_2F	nirKP1_2R	2	1,00	0,90	1,00	1,00	0,89	0,40	1,00	0,00	0,54	0,69	0,48	0,50	0,81	-	-
			3	1,00	1,00	1,00	1,00	0,97	0,80	1,00	0,75	1,00	1,00	1,00	1,00	0,99	-	-
	nirKP1_4F	nirKP1_2R	2	1,00	0,91	1,00	1,00	0,94	0,80	1,00	0,25	0,92	0,75	0,71	1,00	0,93	-	-
			3	1,00	1,00	1,00	1,00	1,00	1,00	1,00	1,00	1,00	1,00	1,00	1,00	1,00	-	-
	nirKP1_10F	nirKP1_1R	2	1,00	0,27	0,18	0,39	0,12	0,20	0,00	0,50	0,15	0,00	0,48	0,29	0,42	-	-
			3	1,00	0,88	1,00	0,75	0,89	1,00	0,86	1,00	0,77	0,69	1,00	1,00	0,91	-	-
	nirKP1_10F	nirKP1_2R	2	1,00	0,87	1,00	1,00	0,90	0,40	0,00	1,00	0,85	0,69	0,71	1,00	0,89	-	-
			3	1,00	1,00	1,00	1,00	0,99	1,00	0,86	1,00	1,00	1,00	1,00	1,00	1,00	-	-
	nirKP1_11F	nirKP1_1R	2	1,00	0,28	0,18	0,39	0,12	0,20	1,00	0,50	0,23	0,00	0,48	0,29	0,44	-	-
			3	1,00	0,88	1,00	0,75	0,90	1,00	1,00	1,00	0,77	0,69	1,00	1,00	0,91	-	-
	nirKP1_11F	nirKP1_2R	2	1,00	0,91	1,00	1,00	0,90	1,00	1,00	1,00	0,92	0,69	0,71	0,71	0,93	-	-
			3	1,00	1,00	1,00	1,00	1,00	1,00	1,00	1,00	1,00	1,00	1,00	1,00	1,00	-	-
Proteobacteria	F1aCu	R3Cu	2	0,57	0,00	0,00	0,00	0,00	0,00	0,00	0,00	0,00	0,00	0,00	0,00	0,14	1,00	0
			3	0,85	0,00	0,00	0,00	0,00	0,00	0,00	0,00	0,00	0,00	0,00	0,00	0,22	1,00	0
	F1aCu	nirK3R	2	0,57	0,00	0,00	0,00	0,00	0,00	0,00	0,00	0,00	0,00	0,00	0,00	0,14	1,00	0
			3	0,70	0,00	0,00	0,00	0,00	0,00	0,00	0,00	0,00	0,00	0,00	0,00	0,18	1,00	0
	F1aCu	nirK5R	2	0,75	0,00	0,00	0,00	0,00	0,00	0,00	0,00	0,00	0,00	0,00	0,00	0,19	1,00	0
			3	0,86	0,00	0,00	0,00	0,00	0,00	0,00	0,00	0,00	0,00	0,00	0,00	0,22	1,00	0
	nirK1F	nirK3R	2	0,41	0,00	0,00	0,00	0,00	0,00	0,00	0,00	0,00	0,00	0,00	0,00	0,10	1,00	0
			3	0,43	0,00	0,00	0,00	0,00	0,00	0,00	0,00	0,00	0,00	0,00	0,00	0,11	1,00	0
	nirK1F	nirK5R	2	0,43	0,00	0,00	0,00	0,00	0,00	0,00	0,00	0,00	0,00	0,00	0,00	0,11	1,00	0
			3	0,47	0,00	0,00	0,00	0,01	0,00	0,00	0,00	0,00	0,00	0,00	0,00	0,12	1,00	1
	nirK1F	R3Cu	2	0,35	0,00	0,00	0,00	0,00	0,00	0,00	0,00	0,00	0,00	0,00	0,00	0,09	1,00	0
			3	0,47	0,00	0,00	0,00	0,00	0,00	0,00	0,00	0,00	0,00	0,00	0,00	0,12	1,00	0
	Cu3F	R3Cu	2	0,69	0,00	0,00	0,00	0,00	0,00	0,00	0,00	0,00	0,00	0,00	0,00	0,17	1,00	0
			3	0,90	0,00	0,00	0,00	0,00	0,00	0,00	0,00	0,00	0,00	0,00	0,00	0,23	1,00	0
	Cu3F	nirK3R	2	0,63	0,00	0,00	0,00	0,00	0,00	0,00	0,00	0,00	0,00	0,00	0,00	0,16	1,00	0
			3	0,70	0,00	0,00	0,00	0,00	0,00	0,00	0,00	0,00	0,00	0,00	0,00	0,18	1,00	0
	Cu3F	nirK5R	2	0,88	0,00	0,00	0,00	0,00	0,00	0,00	0,00	0,00	0,00	0,00	0,00	0,22	1,00	0
			3	0,93	0,00	0,00	0,00	0,00	0,00	0,00	0,00	0,00	0,00	0,00	0,00	0,24	1,00	0
	nirK517F	nirK1055R	2	0,87	0,00	0,00	0,00	0,00	0,00	0,00	0,00	0,00	0,00	0,00	0,00	0,22	1,00	0
			3	0,91	0,00	0,00	0,00	0,00	0,00	0,00	0,00	0,00	0,00	0,00	0,00	0,23	1,00	0
	nirKC1F	nirKC1R	2	0,17	0,00	0,00	0,00	0,00	0,00	0,00	0,00	0,00	0,00	0,00	0,00	0,04	1,00	0
			3	0,39	0,00	0,00	0,00	0,00	0,00	0,00	0,00	0,00	0,00	0,00	0,00	0,10	1,00	0
	F1aCu	nirKP1_8R	2	0,75	0,00	0,00	0,00	0,00	0,00	0,00	0,00	0,00	0,00	0,00	0,00	0,19	1,00	0
			3	0,86	0,00	0,18	0,00	0,00	0,00	0,00	0,00	0,00	0,00	0,00	0,00	0,22	1,00	1
	Cu3F	nirKP1_8R	2	0,83	0,00	0,00	0,00	0,00	0,00	0,00	0,00	0,00	0,00	0,00	0,00	0,21	1,00	0
			3	0,88	0,00	0,27	0,00	0,00	0,00	0,00	0,25	0,00	0,00	0,00	0,00	0,23	1,00	2
	nirKP1_13F	nirKP1_8R	2	0,82	0,00	0,00	0,00	0,00	0,00	0,00	0,00	0,00	0,00	0,00	0,00	0,21	1,00	0
			3	0,88	0,00	0,27	0,00	0,00	0,00	0,00	0,25	0,00	0,00	0,00	0,00	0,23	1,00	2
	nirKP1_10F	nirKP1_8R	2	0,83	0,00	0,00	0,00	0,00	0,00	0,00	0,00	0,00	0,00	0,00	0,00	0,21	1,00	0
			3	0,88	0,00	0,27	0,00	0,00	0,00	0,00	0,25	0,00	0,00	0,00	0,00	0,23	1,00	2
	nirKP1_10F	nirK5R	2	0,89	0,00	0,00	0,00	0,00	0,00	0,00	0,00	0,00	0,00	0,00	0,00	0,22	1,00	0
			3	0,95	0,00	0,00	0,00	0,01	0,00	0,00	0,00	0,00	0,00	0,00	0,00	0,24	1,00	1
	nirKP1_10F	nirK1055R	2	0,89	0,00	0,00	0,00	0,00	0,00	0,00	0,00	0,00	0,00	0,00	0,00	0,22	1,00	0
			3	0,94	0,00	0,00	0,00	0,00	0,00	0,00	0,00	0,00	0,00	0,00	0,00	0,24	1,00	0
	nirKP1_10F	nirKP1_7R	2	0,99	0,01	0,00	0,00	0,00	0,00	0,00	0,00	0,00	0,00	0,00	0,00	0,25	1,00	1
			3	1,00	0,17	0,18	0,25	0,17	0,80	0,29	0,25	0,08	0,06	0,05	0,00	0,38	0,85	10
	nirKP1_4F	nirK1055R	2	0,89	0,00	0,00	0,00	0,00	0,00	0,00	0,00	0,00	0,00	0,00	0,00	0,22	1,00	0
			3	0,94	0,00	0,00	0,00	0,00	0,00	0,00	0,00	0,00	0,00	0,00	0,00	0,24	1,00	0
	nirKP1_4F	nirK5R	2	0,89	0,00	0,00	0,00	0,00	0,00	0,00	0,00	0,00	0,00	0,00	0,00	0,00	1,00	0
			3	0,95	0,00	0,00	0,00	0,01	0,00	0,00	0,00	0,00	0,00	0,00	0,00	0,24	1,00	1
	nirKP1_4F	nirKP1_7R	2	0,99	0,00	0,00	0,00	0,00	0,00	0,00	0,00	0,00	0,00	0,00	0,00	0,25	1,00	0
			3	1,00	0,17	0,18	0,25	0,18	0,80	0,43	0,25	0,08	0,06	0,05	0,00	0,38	0,84	10
AniA	nirKA2_1F	nirKA2_1R	2	0,00	0,84	0,00	0,75	0,19	0,00	0,00	0,00	0,00	0,31	0,57	0,57	0,37	0,91	5
			3	0,00	0,93	0,09	1,00	0,36	0,00	0,00	0,00	0,00	0,56	0,86	0,79	0,47	0,83	6
	nirKA2_2F	nirKA2_1R	2	0,00	0,83	0,00	0,00	0,00	0,00	0,00	0,00	0,00	0,00	0,29	0,43	0,25	0,99	2
			3	0,00	0,88	0,00	0,00	0,00	0,00	0,00	0,00	0,00	0,00	0,76	0,57	0,29	0,99	2
	nirKA2_3F	nirKA2_1R	2	0,00	0,84	0,00	0,00	0,00	0,00	0,00	0,00	0,00	0,00	0,43	0,50	0,26	0,99	2
			3	0,00	0,89	0,00	0,82	0,00	0,00	0,00	0,00	0,00	0,00	0,71	0,64	0,34	0,96	3
	nirKP1_10F	nirKA2_1R	2	0,00	0,80	0,00	0,75	0,15	0,00	0,00	0,00	0,00	0,31	0,57	0,57	0,35	0,92	5
			3	0,00	0,97	0,09	1,00	0,35	0,00	0,00	0,00	0,00	0,56	0,86	0,79	0,48	0,83	6
	nirKP1_8F	nirKA2_1R	2	0,00	0,86	0,00	0,75	0,19	0,00	0,00	0,00	0,00	0,31	0,57	0,57	0,37	0,91	5
			3	0,00	0,97	0,09	1,00	0,36	0,00	0,00	0,00	0,00	0,56	0,86	0,79	0,48	0,83	6
	nirKA2_2F	nirKA2_5R	2	0,00	0,77	0,00	0,00	0,00	0,00	0,00	0,00	0,00	0,00	0,57	0,43	0,24	0,99	2
			3	0,00	0,88	0,00	0,00	0,00	0,80	0,00	0,00	0,00	0,00	0,90	0,57	0,30	0,97	3
	nirKA2_3F	nirKA2_5R	2	0,00	0,78	0,00	0,00	0,00	0,60	0,00	0,00	0,00	0,00	0,86	0,57	0,27	0,98	3
			3	0,00	0,89	0,00	0,00	0,00	0,80	0,00	0,00	0,00	0,00	0,86	0,64	0,30	0,97	3
	nirKA2_3F	nirKA2_6R	2	0,00	0,78	0,00	0,00	0,00	0,00	0,00	0,00	0,00	0,00	0,00	0,07	0,21	1,00	1
			3	0,01	0,83	0,00	0,79	0,00	0,00	0,00	0,00	0,00	0,00	0,10	0,29	0,28	0,97	4
	nirKA2_5F	nirKA2_5R	2	0,00	0,77	0,00	0,00	0,00	0,00	0,00	0,00	0,00	0,00	0,57	0,43	0,24	0,99	2
			3	0,00	0,88	0,00	0,00	0,00	0,80	0,00	0,00	0,00	0,00	0,90	0,57	0,30	0,97	3
	nirKA2_5F	nirKA2_7R	2	0,00	0,76	0,00	0,00	0,00	0,00	0,00	0,00	0,00	0,00	0,57	0,43	0,24	0,99	2
			3	0,00	0,88	0,00	0,00	0,00	0,80	0,00	0,00	0,00	0,00	0,90	0,57	0,30	0,97	3
	nirKA2_5F	nirKA2_6R	2	0,00	0,77	0,00	0,00	0,00	0,00	0,00	0,00	0,00	0,00	0,00	0,00	0,20	1,00	0
			3	0,01	0,83	0,00	0,00	0,00	0,00	0,00	0,00	0,00	0,00	0,10	0,21	0,23	1,00	3
	nirKA2_4F	nirKA2_5R	2	0,00	0,81	0,00	0,00	0,00	0,00	0,00	0,00	0,00	0,00	0,95	0,57	0,28	0,98	2
			3	0,01	0,93	0,00	0,00	0,19	0,80	0,00	0,00	0,08	0,00	0,95	0,64	0,37	0,91	6
	nirKA2_4F	nirKA2_4R	2	0,00	0,71	0,00	0,00	0,00	0,00	0,00	0,00	0,00	0,00	0,00	0,00	0,19	1,00	0
			3	0,00	0,89	0,00	0,00	0,00	0,00	0,00	0,00	0,00	0,00	0,00	0,00	0,24	1,00	0
	nirKP1_10F	nirKA2_8R	2	0,00	0,65	0,00	0,00	0,00	0,00	0,00	0,00	0,00	0,00	0,00	0,00	0,17	1,00	0
			3	0,00	0,89	0,00	0,00	0,00	0,00	0,14	0,00	0,00	0,00	0,00	0,00	0,24	1,00	1
	nirKC2F	nirKC2R	2	0,00	0,12	0,00	0,00	0,00	0,00	0,00	0,00	0,00	0,00	0,00	0,00	0,03	1,00	0
			3	0,00	0,36	0,00	0,00	0,00	0,00	0,00	0,00	0,00	0,00	0,00	0,00	0,09	1,00	0
Haloarchaea	nirKA4_1F	nirKA4_1R	2	0,00	0,00	1,00	0,00	0,00	0,00	0,00	0,00	0,00	0,00	0,00	0,00	0,02	1,00	0
			3	0,00	0,00	1,00	0,00	0,00	0,00	0,00	0,00	0,00	0,00	0,00	0,00	0,02	1,00	0
	nirKA4_1F	nirKA4_2R	2	0,00	0,00	1,00	0,00	0,00	0,00	0,00	0,00	0,00	0,00	0,00	0,00	0,02	1,00	0
			3	0,00	0,00	1,00	0,00	0,00	0,00	0,00	0,00	0,00	0,00	0,00	0,00	0,02	1,00	0
	nirKA4_1F	nirKA4_3R	2	0,00	0,00	0,82	0,00	0,00	0,00	0,00	0,00	0,00	0,00	0,00	0,00	0,02	1,00	0
			3	0,00	0,00	1,00	0,00	0,00	0,00	0,00	0,00	0,00	0,00	0,00	0,00	0,02	1,00	0
	nirKA4_2F	nirKA4_1R	2	0,00	0,00	1,00	0,00	0,00	0,00	0,00	0,00	0,00	0,00	0,00	0,00	0,02	1,00	0
			3	0,00	0,00	1,00	0,00	0,01	0,20	0,00	0,00	0,00	0,00	0,00	0,00	0,03	1,00	2
	nirKA4_2F	nirKA4_2R	2	0,00	0,00	0,30	0,00	0,00	0,00	0,00	0,00	0,00	0,00	0,00	0,00	0,02	1,00	0
			3	0,00	0,00	1,00	0,00	0,01	0,00	0,00	0,00	0,00	0,00	0,00	0,00	0,03	1,00	1
	nirKA4_2F	nirKA4_3R	2	0,00	0,00	0,82	0,00	0,00	0,00	0,00	0,00	0,00	0,00	0,00	0,00	0,02	1,00	0
			3	0,00	0,00	1,00	0,00	0,01	0,00	0,00	0,00	0,00	0,00	0,00	0,00	0,03	1,00	1
	nirKA4_3F	nirKA4_1R	2	0,00	0,00	1,00	0,00	0,00	0,00	0,00	0,00	0,00	0,00	0,00	0,00	0,02	1,00	0
			3	0,00	0,00	1,00	0,00	0,00	0,00	0,00	0,00	0,00	0,00	0,00	0,00	0,02	1,00	0
	nirKA4_3F	nirKA4_2R	2	0,00	0,00	1,00	0,00	0,00	0,00	0,00	0,00	0,00	0,00	0,00	0,00	0,02	1,00	0
			3	0,00	0,00	1,00	0,00	0,00	0,00	0,00	0,00	0,00	0,00	0,00	0,00	0,02	1,00	0
	nirKA4_3F	nirKA4_3R	2	0,00	0,00	0,82	0,00	0,00	0,00	0,00	0,00	0,00	0,00	0,00	0,00	0,02	1,00	0
			3	0,00	0,00	1,00	0,00	0,00	0,00	0,00	0,00	0,00	0,00	0,00	0,00	0,02	1,00	0
Actinobacteria	nirKA6_1F	nirKA6_1R	2	0,00	0,00	0,00	0,00	0,85	0,00	0,00	0,00	0,00	0,00	0,00	0,00	0,19	1,00	0
			3	0,03	0,01	0,00	0,00	0,96	0,00	0,00	0,00	0,00	0,00	0,10	0,07	0,23	0,99	4
	nirKA6_1F	nirKA6_2R	2	0,00	0,00	0,00	0,00	0,81	0,00	0,00	0,00	0,00	0,00	0,00	0,00	0,18	1,00	0
			3	0,00	0,06	0,00	0,00	0,93	0,00	0,00	0,00	0,00	0,00	0,00	0,14	0,22	1,00	2
	nirKA6_1F	nirKA6_3R	2	0,00	0,00	0,00	0,00	0,81	0,00	0,00	0,00	0,00	0,00	0,00	0,00	0,18	1,00	0
			3	0,00	0,00	0,00	0,00	0,96	0,00	0,00	0,00	0,00	0,00	0,00	0,00	0,21	1,00	0
	nirKC3F	nirKC3R	2	0,00	0,00	0,00	0,00	0,06	0,00	0,00	0,00	0,00	0,00	0,00	0,00	0,01	1,00	0
			3	0,00	0,00	0,00	0,00	0,11	0,00	0,00	0,00	0,00	0,00	0,00	0,00	0,02	1,00	0
Hyphomicrobia	nirKH3_1F	nirKH3_1R	2	0,00	0,00	0,00	0,00	0,00	0,00	0,00	0,00	0,00	0,00	0,00	0,00	0,00	1,00	0
			3	0,00	0,00	0,00	0,00	0,01	0,00	0,00	0,00	0,00	0,00	0,00	0,00	0,00	1,00	1
	Cu3F	nirKH3_1R	2	0,00	0,22	0,00	0,04	0,00	0,00	0,00	0,00	0,00	0,00	0,81	0,29	0,11	0,98	3
			3	0,01	0,33	0,00	0,29	0,00	0,00	0,00	0,00	0,00	0,00	1,00	0,57	0,17	0,95	4
	nirKP1_10F	nirKH3_1R	2	0,00	0,37	0,00	0,54	0,00	0,00	0,00	0,00	0,00	0,00	1,00	0,29	0,19	0,96	3
			3	0,01	0,58	0,00	0,54	0,02	0,00	0,00	0,00	0,00	0,00	1,00	0,57	0,26	0,90	5
	nirKP1_11F	nirKH3_1R	2	0,00	0,38	0,00	0,54	0,00	0,00	0,00	0,00	0,00	0,00	1,00	0,29	0,19	0,96	3
			3	0,01	0,58	0,00	0,54	0,02	0,00	0,00	0,00	0,00	0,00	1,00	0,57	0,26	0,90	5
	nirKP1_13F	nirKH3_1R	2	0,00	0,26	0,00	0,29	0,00	0,00	0,00	0,00	0,00	0,00	0,95	0,29	0,14	0,97	3
			3	0,01	0,49	0,00	0,36	0,00	0,00	0,00	0,00	0,00	0,00	1,00	0,57	0,22	0,93	4
	nirKP1_13F	NirK-R	2	0,00	0,03	0,00	0,00	0,00	0,00	0,00	0,00	0,00	0,00	0,43	0,29	0,04	1,00	2
			3	0,00	0,06	0,00	0,14	0,00	0,00	0,00	0,00	0,00	0,00	0,57	0,43	0,06	0,99	3
	nirKA4_2F	nirKH3_1R	2	0,00	0,09	0,00	0,04	0,00	0,00	0,00	0,00	0,00	0,00	0,57	0,29	0,06	0,99	3
			3	0,00	0,23	0,00	0,21	0,00	0,00	0,00	0,00	0,00	0,00	0,86	0,57	0,13	0,97	3
	nirKH3_2F	nirKH3_3R	2	0,00	0,27	0,00	0,00	0,00	0,00	0,00	0,00	0,00	0,00	0,95	0,29	0,12	0,98	2
			3	0,00	0,43	0,09	0,21	0,00	0,00	0,00	0,00	0,00	0,00	1,00	0,57	0,19	0,94	4
	nirKH3_2F	nirKH3_2R	2	0,00	0,16	0,00	0,00	0,00	0,00	0,00	0,00	0,00	0,00	0,86	0,21	0,09	0,99	2
			3	0,00	0,33	0,00	0,00	0,00	0,00	0,00	0,00	0,00	0,00	0,95	0,50	0,15	0,98	2
Nitrosomonas	nirKN7_1F	nirKN7_3R	2	0,00	0,00	0,00	0,00	0,00	0,00	0,00	0,00	0,69	0,00	0,00	0,00	0,02	1,00	0
			3	0,00	0,00	0,00	0,04	0,01	0,00	0,00	0,00	0,77	0,00	0,00	0,00	0,03	1,00	2
	nirKH7_2F	nirKN7_3R	2	0,00	0,00	0,00	0,04	0,01	0,00	0,00	0,00	0,77	0,00	0,00	0,00	0,03	1,00	0
			3	0,00	0,00	0,00	0,00	0,00	0,00	0,00	0,00	0,77	0,00	0,00	0,00	0,02	1,00	1
	nirKH7_3F	nirKN7_3R	2	0,00	0,00	0,00	0,00	0,00	0,00	0,00	0,00	0,77	0,00	0,00	0,00	0,02	1,00	0
			3	0,00	0,00	0,00	0,00	0,00	0,00	0,00	0,00	0,85	0,00	0,00	0,00	0,02	1,00	0
	nirKH7_1F	nirKN7_4R	2	0,00	0,00	0,00	0,00	0,00	0,00	0,00	0,00	0,85	0,00	0,00	0,00	0,02	1,00	0
			3	0,00	0,05	0,00	0,00	0,00	0,00	0,00	0,00	0,69	0,00	0,00	0,00	0,02	0,98	4
	nirKH7_2F	nirKN7_4R	2	0,00	0,00	0,00	0,14	0,07	0,00	0,00	0,00	0,77	0,00	0,00	0,21	0,07	1,00	0
			3	0,02	0,00	0,00	0,00	0,00	0,00	0,00	0,00	0,77	0,00	0,00	0,00	0,02	1,00	2
	nirKH7_3F	nirKN7_4R	2	0,00	0,00	0,00	0,00	0,02	0,00	0,00	0,00	0,85	0,00	0,00	0,00	0,03	1,00	0
			3	0,00	0,00	0,00	0,00	0,00	0,00	0,00	0,00	0,77	0,00	0,00	0,00	0,02	1,00	1
	nirKH7_1F	nirKN7_2R	2	0,00	0,00	0,00	0,00	0,02	0,00	0,00	0,00	0,85	0,00	0,00	0,00	0,03	1,00	0
			3	0,00	0,00	0,00	0,00	0,00	0,00	0,00	0,00	0,54	0,00	0,00	0,00	0,02	1,00	0
	nirKH7_2F	nirKN7_2R	2	0,00	0,00	0,00	0,00	0,00	0,00	0,00	0,00	0,77	0,00	0,00	0,00	0,02	1,00	0
			3	0,00	0,00	0,00	0,00	0,00	0,00	0,00	0,00	0,54	0,00	0,00	0,00	0,02	1,00	0
	nirKH7_3F	nirKN7_2R	2	0,00	0,00	0,00	0,00	0,00	0,00	0,00	0,00	0,77	0,00	0,00	0,00	0,02	1,00	0
			3	0,00	0,00	0,00	0,00	0,00	0,00	0,00	0,00	0,54	0,00	0,00	0,00	0,02	1,00	0
	nirKH7_1F	nirKN7_1R	2	0,00	0,00	0,00	0,00	0,00	0,00	0,00	0,00	0,77	0,00	0,00	0,00	0,02	1,00	0
			3	0,00	0,00	0,00	0,00	0,00	0,00	0,00	0,00	0,54	0,00	0,00	0,00	0,02	1,00	0
	nirKH7_2F	nirKN7_1R	2	0,00	0,00	0,00	0,00	0,00	0,00	0,00	0,00	0,77	0,00	0,00	0,00	0,02	1,00	0
			3	0,00	0,00	0,00	0,00	0,00	0,00	0,00	0,00	0,54	0,00	0,00	0,00	0,02	1,00	0
	nirKH7_3F	nirKN7_1R	2	0,00	0,00	0,00	0,00	0,00	0,00	0,00	0,00	0,77	0,00	0,00	0,00	0,02	1,00	0
			3	0,00	0,00	0,00	0,00	0,00	0,00	0,00	0,00	0,54	0,00	0,00	0,00	0,02	1,00	0
	nirKC4F	nirKC4R	2	0,00	0,00	0,00	0,00	0,00	0,00	0,00	0,00	0,08	0,00	0,00	0,00	0,00	1,00	0
			3	0,00	0,00	0,00	0,00	0,00	0,00	0,00	0,00	0,15	0,00	0,00	0,00	0,00	1,00	0
Eukaryotes	nirKfF	nirKfR	2	0,00	0,00	0,00	0,14	0,00	0,00	0,00	0,00	0,00	0,00	0,00	0,00	0,01	1,00	0
			3	0,00	0,00	0,00	0,29	0,00	0,00	0,00	0,00	0,00	0,00	0,00	0,00	0,02	1,00	0
	EunirK-F1	EunirK-R1	2	0,00	0,00	0,00	0,29	0,00	0,00	0,00	0,00	0,00	0,00	0,00	0,00	0,02	1,00	0
			3	0,00	0,00	0,09	0,86	0,00	0,00	0,00	0,00	0,00	0,00	0,00	0,00	0,05	1,00	1
Firmicutes	GnirK2F	GnirK2R	2	0,00	0,00	0,00	0,00	0,00	0,00	0,00	0,00	0,00	0,56	0,00	0,00	0,00	1,00	0
			3	0,00	0,00	0,00	0,00	0,00	0,00	0,00	0,00	0,00	0,63	0,00	0,00	0,00	1,00	0

Table S4. Metagenomes from where *nirS* and *nirK* sequences were retrieved. They are grouped by environment and ecosystem categories as reported in IMG.

Environment	Ecosystem Category	Ecosystem Subtype	Study Name	Sample Name	taxon_oid	IMG Genome ID	Is Public	Genome Size (bp)	Gene Count
**Freshwater**	Aquatic	Groundwater	Groundwater microbial communities from subsurface biofilms in sulfidic aquifier in Frasassi Gorge, Italy	AS07_7	3300000227	3300000227	Yes	300 437 395	**538 069**
				FS06_10	3300000233	3300000233	Yes	242 610 534	**471 920**
				FS08_3	3300000236	3300000236	Yes	181 303 868	**400 347**
				GS09_5	3300000234	3300000234	Yes	539 638 524	**1 025 572**
				LI09_3	3300000229	3300000229	Yes	281 841 843	**721 213**
				PC08_3	3300000235	3300000235	Yes	252 007 248	**513 563**
				PC08_64	3300000232	3300000232	Yes	270 071 044	**568 490**
				PC08_66	3300000228	3300000228	Yes	448 373 019	**974 097**
			Groundwater microbial communities from Rifle, Colorado - Rifle Oxygen_injection	A2	3300002146	3300002146	Yes	118 930 077	**269 038**
				A3	3300002152	3300002152	Yes	200 118 367	**441 302**
				B2	3300002096	3300002096	Yes	121 439 435	**277 091**
				B3	3300002094	3300002094	Yes	63 701 458	**157 562**
				C3	3300002095	3300002095	Yes	73 108 706	**174 109**
		Lentic	Freshwater and sediment microbial communities from a dead zone in Lake Erie	Lake Erie CCB hypolimnion	3300000756	3300000756	Yes	387 873 697	**897 169**
			Freshwater microbial communities from Trout Bog Lake, WI and Lake Mendota, IL	Trout Bog Hypolimnion	3300000553	3300000553	Yes	547 080 431	**690 501**
			Freshwater sediment microbial communities from Lake Washington, Seattle, USA, for methane and nitrogen cycles	(Flow sorted aerobic no nitrate)	2084038009	2084038009	Yes	396 601 732	**1 059 847**
				(Aerobic without added nitrate, 13C SIP)	2046860007	2046860007	Yes	76 631 179	**210 153**
				(Aerobic with added nitrate, 13C SIP)	2046860006	2046860006	Yes	80 246 742	**216 380**
				(Anaerobic + nitrate SIP Nov 2010 with PE)	2088090009	2088090009	Yes	354 011 745	**948 029**
				(Aerobic without added nitrate, 13C SIP Nov 2010 with PE)	2088090013	2088090013	Yes	308 706 277	**821 124**
				(Original sample replicate 1)	2088090005	2088090005	Yes	559 537 102	**1 554 712**
				(Flow sorted aerobic plus nitrate)	2100351007	2100351007	Yes	386 453 037	**1 065 361**
				(Flow sorted anaerobic no nitrate)	2140918012	2140918012	Yes	47 869 084	**96 914**
				(Aerobic without added nitrate, SIP additional fraction)	2046860005	2046860005	Yes	58 805 457	**157 525**
			Sediment methylotrophic communities from Lake Washington	Methane enrichment	2006543005	2006543005	Yes	211 470 570	**323 777**
				Methanol enrichment	2006207001	2006207001	Yes	50 245 961	**77 750**
				Methylamine enrichment	2006207002	2006207002	Yes	37 225 208	**54 783**
**Soil**	Soil	Terrestrial	Soil microbial communities from Great Prairies (Kansas, Wisconsin and Iowa)	Iowa, Continuous Corn soil (gaii)	2228664021	2228664021	Yes	647968790	**1 499 399**
				Iowa, Continuous Corn soil (Illumina)	2088090015	2088090015	Yes	244 906 069	**353 311**
				Iowa, Native Prairie soil (MSU Illumina)	2088090014	2088090014	Yes	279 478 596	**400 426**
				Iowa, Native Prairie soil (MSU hiseq+gaii)	3300000789	3300000789	Yes	1 503 004 606	**4 175 735**
				Kansas Corn soil	2067725004	2067725004	Yes	334 907 586	**941 175**
				Kansas, Cultivated Corn soil (JGI Velvet)	3300000953	3300000953	Yes	1 342 147 687	**5 393 094**
				Kansas, Native Prairie soil	2070309009	2070309009	Yes	375 620 835	**1 065 090**
				Wisconsin, Continuous corn soil (JGI Velvet)	3300000891	3300000891	Yes	1 463 277 025	**4 632 915**
			Soil microbial communities from Great Prairies (Kansas, Wisconsin and Iowa)	Wisconsin Native Prairie soil (JGI Velvet)	3300000858	3300000858	Yes	581 758 822	**2 215 907**
				Wisconsin Restored Prairie soil	2067725000	2067725000	Yes	149 161 609	**409 243**
				Wisconsin Restored Prairie soil (JGI Velvet)	3300000881	3300000881	Yes	259 370 859	**706 571**
				Wisconsin, Switchgrass soil	2067725003	2067725003	Yes	123 787 058	**337 861**
**Wastewater**	Industrial wastewater	Petrochemical	Hydrocarbon resource environments microbial communities from Canada and USA	Coal-degrading lab enrichment, Bowden, Alberta	3300000507	3300000507	Yes	28 712 273	**40 166**
				Oil fields, Alberta, Canada - degrading Toluene	3300001567	3300001567	Yes	605 635 502	**1 184 637**
				Tailings pond Northern Alberta -methanogenic enrichment culture SCADC	3300000568	3300000568	Yes	646 672 477	**1 513 645**
				Tailings pond Northern Alberta - Syncrude Mildred Lake Settling Basin	3300001605	3300001605	Yes	1 619 957 270	**3 740 874**
				Tailings pond Northern Alberta -TP6_2008_2010:	2209111015	2209111015	Yes	232 102 284	**638 670**
				Syncrude, Ft. McMurray, Alberta - Suncor 6 2012TP6_6	3300001580	3300001580	Yes	1 282 178 941	**3 205 687**
				Syncrude, Ft. McMurray, Alberta - Medicine Hat oil field -PW_MHGC_2012April2:	3300001592	3300001592	Yes	1 015 936 974	**2 251 644**
				Syncrude, Ft. McMurray, Alberta - West In Pit SyncrudeMLSB2011	3300000558	3300000558	Yes	1 148 076 561	**3 021 632**

References

1. Casciotti, K. L. & Ward, B. B. Dissimilatory nitrite reductase genes from autotrophic ammonia-oxidizing bacteria. *Appl. Environ. Microbiol.* **67,** 2213–21 (2001).

2. Hallin, S. & Lindgren, P.-E. PCR Detection of Genes Encoding Nitrite Reductase in Denitrifying Bacteria. *Appl. Envir. Microbiol.* **65,** 1652–1657 (1999).

3. Braker, G., Fesefeldt, A. & Witzel, K.-P. Development of PCR Primer Systems for Amplification of Nitrite Reductase Genes (nirK and nirS) To Detect Denitrifying Bacteria in Environmental Samples. *Appl. Envir. Microbiol.* **64,** 3769–3775 (1998).

4. Chen, Z. *et al.* Impact of long-term fertilization on the composition of denitrifier communities based on nitrite reductase analyses in a paddy soil. *Microb. Ecol.* **60,** 850–61 (2010).

5. Wei, W. *et al.* Higher diversity and abundance of denitrifying microorganisms in environments than considered previously. *ISME J.* (2015). doi:10.1038/ismej.2015.9

6. Green, S. J. *et al.* Denitrifying bacteria isolated from terrestrial subsurface sediments exposed to mixed-waste contamination. *Appl. Environ. Microbiol.* **76,** 3244–54 (2010).

7. Maeda, K. *et al.* N2O production, a widespread trait in fungi. *Sci. Rep.* **5,** 9697 (2015).

8. Wei, W. *et al.* Development of PCR primers targeting fungal nirK to study fungal denitrification in the environment. *Soil Biol. Biochem.* **81,** 282–286 (2015).

9. Verbaendert, I., Hoefman, S., Boeckx, P., Boon, N. & De Vos, P. Primers for overlooked nirK, qnorB, and nosZ genes of thermophilic Gram-positive denitrifiers. *FEMS Microbiol. Ecol.* **89,** 162–80 (2014).

10. Michotey, V., Méjean, V. & Bonin, P. Comparison of methods for quantification of cytochrome cd(1)-denitrifying bacteria in environmental marine samples. *Appl. Environ. Microbiol.* **66,** 1564–71 (2000).

11. Throbäck, I. N., Enwall, K., Jarvis, A. & Hallin, S. Reassessing PCR primers targeting nirS, nirK and nosZ genes for community surveys of denitrifying bacteria with DGGE. *FEMS Microbiol. Ecol.* **49,** 401–17 (2004).
